# Prediction of Purchase Volume of Cross-Border e-Commerce Platform Based on BP Neural Network

**DOI:** 10.1155/2022/3821642

**Published:** 2022-04-15

**Authors:** Xiang Zhang

**Affiliations:** Zhuhai College of Science and Technology, Zhuhai, Guangdong 519040, China

## Abstract

As a new form of foreign trade, cross-border e-commerce has huge development potential. Although the development prospect of cross-border e-commerce is good, the management of global supply chain is very important in order to gain a place in the fierce competition and develop steadily. The traditional forecasting of purchasing volume adopts time series, and the forecasting model is relatively simple. The purchase volume of the platform is related to the various consumption behaviors of consumers, such as the number of product reviews, the number of product collections, and whether there are tax subsidies. The sales volume in the next few days is predicted by the item number, time, and sales quantity. The four-layer BP neural network model is used, and the MATLAB neural network toolbox is used to draw the training error curve and the correlation coefficient curve. After network training, the training correlation coefficient *R* reaches 95.823%, and the prediction accuracy obtained at this time is higher. Further, using the established model based on BP algorithm, the traditional BP algorithm is optimized to obtain the purchase quantity of commodities. The method is applied to the forecast of commodity purchase volume of a cross-border e-commerce platform, and the results show that the average error rate of this method is 5.9%, which has high practical application value. The research results show that this paper considers multiple influencing factors and selects an appropriate forecasting method, which can effectively improve the accuracy of the company's commodity sales forecast, so as to better formulate procurement plans and optimize inventory structure, which has certain implications for the actual operation of cross-border e-commerce platforms.

## 1. Introduction

**Figure 1:**
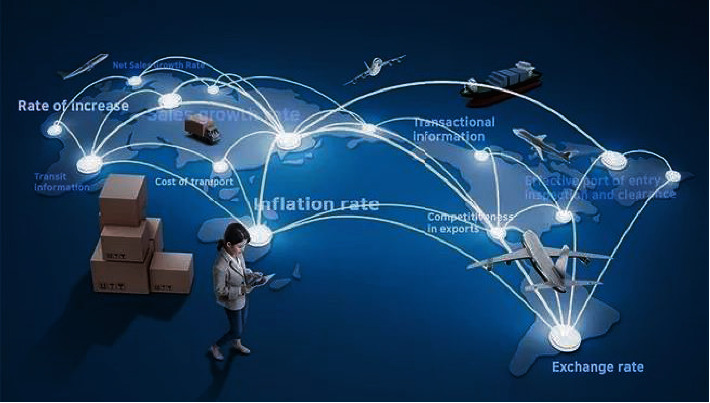
Cross-border e-commerce.

Today, with the development of information technology, people have gradually entered the information age, and traditional product sales methods have been unable to meet people's shopping needs. In the e-commerce work, no matter the size of the seller's store, the problem that the seller must be unable to avoid is the replenishment of orders, and the two main problems faced by the replenishment of orders are the occupation of cash flow and the problem of inventory. If the replenishment order is not predicted well, the capital occupation will have a very large impact on the capital operation of the merchant, and the backlog of inventory will also increase the storage cost and even cause the capital flow to slow down. In addition, this paper selects cross-border e-commerce platforms (Figure 1) to model and predict sales, which further narrows the scope of the study. This idea provides a new perspective for studying cross-border e-commerce platform purchases. In addition, when selecting influencing factors, variable indicators related to the platform itself are introduced, which enrich the types of factors currently used to study the purchase volume of cross-border e-commerce platforms. By comparing the forecasting results of different models, a sales forecasting model with higher forecasting accuracy that is different from the traditional sales forecasting method is determined. On the other hand, by constructing an appropriate sales volume forecast model and forecasting sales volume, the e-commerce platform can accurately formulate policies and marketing strategies that are consistent with the current economic environment and the development of the e-commerce market according to the forecast results and promote the e-commerce platform: healthy and stable development. At the same time, it also helps the company's regulatory department to monitor the company's production capacity in real time, arrange production plans reasonably, avoid the problem of excess production capacity, and help the company to reduce inventory costs and reduce marketing pressure. Therefore, it is of positive significance to accurately predict the purchase volume of cross-border e-commerce platforms [1–5].

Qualitative forecasting method, also known as empirical judgment method, is mainly used by forecasters to estimate and judge the future development trend of the market environment based on personal knowledge and experience, plus various information obtained through market research. Qualitative prediction method has obvious advantages for start-ups, it is time consuming, simple, and easy to operate and can comprehensively consider a variety of factors. The disadvantage is that it is subjective and arbitrary, and the forecast results are not accurate enough. It is more suitable for long-term market environment forecasting than short-term forecasting. Commonly used qualitative prediction methods include comprehensive opinion method, subjective probability method, expert evaluation method, and Delphi method. Qualitative prediction does not need to analyze a large amount of data and does not require relevant mathematical statistics. It only relies on professionals' knowledge of the industry and their grasp of the actual situation to make judgments [6–10].

Compared with qualitative forecasting, quantitative forecasting abandons most of the subjective factors and no longer highly depends on the individual ability of the forecaster. At present, quantitative forecasting methods are more widely used. Quantitative prediction excludes the subjective experience of people, and to a greater extent, on the basis of facts and data, it is necessary to find more historical data, and use computers to establish data models to explore the potential laws in the data to calculate the prediction results. In short-term forecasting, quantitative forecasting is more accurate than qualitative forecasting, but it requires certain professional skills for forecasters. At present, quantitative forecasting is mainly divided into time series forecasting method, causality forecasting method, and other forecasting forecasting methods [11–15].

Data mining forecasting mainly includes the following three parts: algorithms and techniques, data, and modeling. Among them, the selection and optimization of prediction algorithms and techniques are the top priorities of experts' research in recent years. The research focus of this paper is based on the problem of forecasting the purchase volume of cross-border e-commerce platforms. Through comparison, the algorithm model with the best prediction effect is selected to complete the forecast of cross-border e-commerce platform purchase volume. First, a suitable prediction algorithm needs to be determined. As an important link in the data mining forecasting process, the selection of the forecasting algorithm also prepares for the subsequent accurate forecast of the purchase volume of the cross-border e-commerce platform. Machine learning prediction algorithm is a commonly used prediction algorithm in data mining prediction analysis. The following will focus on the basic principles and advantages and disadvantages of several machine learning prediction algorithms and choose the most suitable algorithm to complete the prediction after optimization [16–19].

Machine learning is a technology that has emerged in recent years and has been widely used in many fields, such as data mining, computer vision, natural language processing, biometric recognition, search engines, medical diagnosis, stock market analysis, and DNA sequence testing. Assuming that there is a functional relationship *y* = *f*(*xB*) between the input and output of things, where *y* is the undetermined parameter and *x* is the input variable, then *y* = *f*(*xB* is called a learning machine. Through modeling, the model is repeatedly trained by the sample data to obtain the value of parameter B, and the functional relationship between *x* and *y* in *y* = *f*(*xB* is determined, so that the *y* value of the new *x* can be predicted. This process is a typical machine learning process. Among them, the classic machine learning prediction methods include decision tree method, support vector machine, Bayesian network, time series model, neural network, and so on [20–22].

There are many kinds of forecasting methods commonly used at present, mainly including time series forecasting method, smoothing forecasting method, trend line forecasting method, seasonal cycle forecasting method, regression forecasting technique, and so on. Although these methods have been applied in various occasions, they have the following deficiencies more or less: (1) most of the models can only be applied to linear situations and cannot be applied well to nonlinear situations; (2) it is difficult to apply to multifactor situations; and (3) the establishment of the model depends on the forecaster's understanding of specific problems and experience in forecasting.

Artificial neural network is a nonlinear system that simulates the information processing method of the human brain. It has the characteristics of distributed storage and parallel processing of knowledge and has the functions of memory and association. Theoretical research shows that it can approximate any nonlinear function with arbitrary precision, so it is more suitable for modeling some complex problems. Prediction of sales volume is undoubtedly very important for operators, but there are many factors that affect sales volume. Under the current situation in my country, it is also related to many national policies, etc., and it is difficult to accurately predict with conventional methods. To this end, this paper discusses the use of neural networks to establish a sales forecast model, uses the memory ability of neural networks to learn and memorize historical data, establishes a neural network model of sales, and then uses the associative ability of neural networks to predict future sales [23–25].

## 2. BP Neural Network

**Figure 2:**
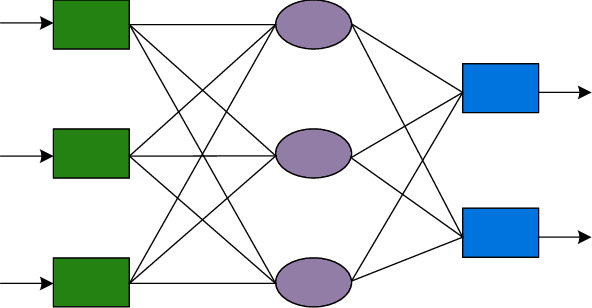
The three-layer network structure diagram.

BP neural network is a multilayer feed-forward network, which has been developed so far and is one of the most widely used neural network models in various industries. Its network topology consists of an input layer, several hidden layers, and an output layer. Among them, the three-layer network structure diagram is shown in Figure 2. BP neural network has complex classification ability and strong nonlinear modeling and mapping ability and is widely used in complex prediction problems. The BP neural network can continuously repair the error between the expectation and the sample data, which is derived from its calculation and adjustment of its own weight threshold to make corrections. However, this model also has certain problems. The BP algorithm has a slow convergence speed, and the BP algorithm is easy to fall into local minimization.

There are many types of neural network structures. This paper adopts a commonly used forward neural network model to solve the problem of sales forecasting. The most commonly used learning algorithm for the forward neural network is the BP algorithm, but the BP algorithm is easy to fall into the local minimum. Evolutionary algorithm is an optimization method based on group optimization. Its basic idea is to simulate the evolution process of biological groups in nature. According to Darwin's evolutionary thought, in nature, biological groups compete with each other and proceed according to the principle of survival of the fittest. Selection of individuals who adapt to the environment is retained, and individuals who do not adapt to the environment are eliminated. Individuals can inherit the characteristics of their parents, and at the same time mutate to have different characteristics from their parents, which provides more and different individuals for selection in nature. This is how the group evolves from generation to generation, and finally evolves from being incompatible with the environment to adapting to the environment. Generally, there are three different types of evolutionary algorithms: genetic algorithms, evolutionary programming, and evolutionary strategies. Due to the characteristics of neural network learning, the learning of evolutionary strategies has certain advantages. The specific steps of the BP algorithm can be simply summarized as follows:

1. Given the input vector *X* and target output vector *T* of the network and initialize the neural network weights;
2. Calculate the actual output of the network;
3. Calculate the error between the actual output vector of the network and the required target output value;
4. Weight learning to minimize the error;

Then repeat steps (1)–(4) to minimize the systematic error.

In the training process of BP neural network, in order to reduce the error between the expected value and the actual value, each connection weight is corrected layer by layer according to the reverse direction of the forward propagation process. It is called the error back-propagation algorithm. With the continuous correction of error back-propagation training, the correct rate of the network's response to the input mode will also continue to improve. The whole process includes forward propagation and back propagation. These two processes are described in detail below:

Step 1: forward propagation process. Let the weight between node *i* and node *j* be *W*_*ij*_, the threshold of node *j* is *b*, and *x* is the output value of the node. Substitute *w*_*ij*_ and *b*_*j*_ into the activation function to obtain the output value of each node according to the following formula:
  (1) $$\begin{array}{c} S_{j}=\sum_{i=0}^{m-1}w_{ij}x_{i}+b_{j},\\ x_{j}=f\left(S_{j}\right), \end{array}$$
where *f* is the activation function.
Step 2: backpropagation process. In the reverse direction of forward propagation, the output layer passes through the hidden layer, and finally returns to the input layer to correct the weights and thresholds of each neuron layer by layer until the error between the expected value and the predicted value reaches the minimum. Assuming that the result of the output layer is *d*_*j*_ and the expected output is *y*_*j*_, the error function is as follows:
  (2) $$\begin{array}{c} E\left(w,b\right)=\frac{1}{2}\sum_{j=0}^{n-1}{\left(d_{j}-y_{j}\right)}^{2}. \end{array}$$

It can be seen from the gradient descent method adopted by the traditional BP neural network that the correction value of the weight is proportional to the gradient of *E*(*w*, *b*), then for the *j*th output node is

(3) $$\begin{array}{c} \Delta w\left(i,j\right)=\eta \frac{\partial E\left(w,b\right)}{\partial w\left(i,j\right)}, \end{array}$$

where *η* is the learning rate. If the activation function is selected as

(4) $$\begin{array}{c} f\left(x\right)=\frac{A}{1+e^{x/B}}. \end{array}$$

That is, the weights and thresholds between the hidden layer and the output layer are adjusted according to the following formula:

(5) $$\begin{array}{c} w_{ij}=w_{ij}-\eta_{1}\delta_{ij}x_{i},\\ b_{j}=b_{j}-\eta_{2}\delta_{ij}. \end{array}$$

Similarly, according to the above method, the *w*_*ij*_ and *b*_*j*_ between the input layer and the hidden layer are adjusted as follows:

(6) $$\begin{array}{c} w_{ki}=w_{ki}-\eta_{1}\delta_{ki}x_{k},\\ b_{i}=b_{i}-\eta_{2}\delta_{ki}. \end{array}$$

**Figure 3:**
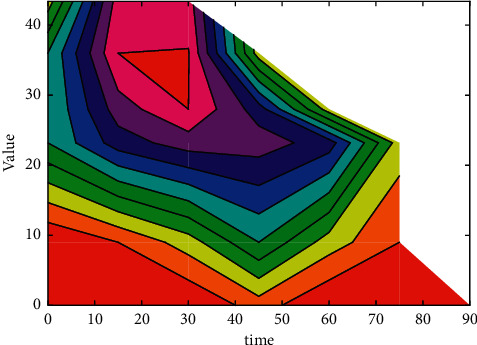
Predicted value.

Among them, *η* is the learning rate, *δ* is the local gradient, and *x*_*k*_ is the output signal of the previous layer. The predicted value is shown in Figure 3.

Once the representation of the neural network is determined, the next step in evolutionary learning is to generate the neural network by randomly generating strings. The performance index of the neural network is related to the cumulative error sum of squares:

(7) $$\begin{array}{c} f=F_{\max}-\sum_{i=1}^{N}\left(y_{i}-{\hat{y}}_{i}\right). \end{array}$$

In the formula, *y*_*i*_ is the expected output, which comes from the sample (*x*_*i*_, *y*_*i*_); *y* is the actual output of the neural network when the input is *x*_*i*_; and *F*_max_ is a preset coefficient. The larger the *f* is, the closer the neural network is to the sample point, so its corresponding performance is better.

Different from the genetic algorithm, the evolution strategy does not use the crossover operator, it only uses the mutation operator, which can mutate the topology and weights of the neural network at the same time. For simplicity, this paper assumes that the topology of the neural network is fixed (using forward neural network, the number of neurons in each layer is fixed), so it is only necessary to mutate the weights. The weight mutation is to add an increment to the original weights. For the weight of the nth neural network in the group, the increment is

(8) $$\begin{array}{c} \Delta w_{ij}^{k}=N\left(0,p_{m}\right),\\ p_{m}=\lambda \left(\frac{1-f}{f_{\max}}\right). \end{array}$$

In the formula, *λ* is the coefficient, *N*(0, *p*_*m*_) is a Gaussian function with a mean of 0 and a variance of *p*_*m*_, and *p*_*m*_ is related to the relative performance of the nth neural network in the population, if the performance of the nth neural network is worse, the greater the pm, the greater the possibility that Δ*w* deviates from the mean value of 0.

**Figure 4:**
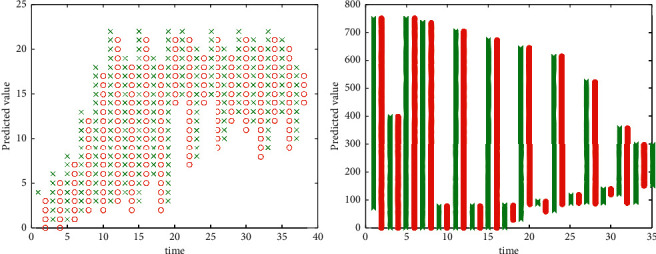
Predicted data.

From the learning process of the evolution strategy to the neural network, it can be seen that the evolution strategy adopts the method of group learning, so it can avoid falling into the local extreme point because it only uses the mutation operator, if necessary, it can the topology of the neural network is learned. The predicted data are shown in Figure 4.

## 3. BP Neural Network Modeling

When analyzing the influencing factors of the purchase volume of cross-border e-commerce platforms, this paper selects the desensitization data set of food, cosmetics, and clothing from a self-operated cross-border e-commerce platform from January 2003 to September 2018. A total of 13 influencing factor indicators were selected. Through correlation analysis and gray correlation analysis, it was found that the selected factors and indicators had high correlation and correlation with the procurement of cross-border e-commerce platforms with a lag of one period. Therefore, when building the BP neural network model, refer to the analysis results, and still select the number of reviews, unit price, good rating, 7-day return, season, tax available, discount available, inventory, purchase rate, number of items added to the shopping cart, sales, and items. The neural network prediction model is constructed by 13 indicators such as type, number of advertisement-guided views, and so on. Since the measurement units of these influencing factors are different and the value ranges are very different, the data also need to be standardized before constructing the BP neural network model.

After determining the variables that affect the purchase volume of the cross-border e-commerce platform, divide the collected data into training data and test data. The division method is consistent with the construction of the linear regression model, where the output variable (*Y*) is the cross-border e-commerce lag period by one period. The input variable (*X*) is the 13 influencing factors that are highly correlated and correlated with the purchase volume of cross-border e-commerce platforms selected in this paper. Due to the small amount of data, 80% of the data is divided into training data and the remaining 20% is used as test data. The data of various influencing factors from 2017 to 2019 are compared with the purchase volume data of cross-border e-commerce platforms from 2017 to 2018. The combination is used as the data for the training model, and the data of each influencing factor from 2018 to 2019 and the purchase volume data of the cross-border e-commerce platform from 2017 to 2019 are combined as the data for the test model. After the data are divided, the BP neural network model is constructed by using the standardized data of each index. The details are as follows:

1. Determine the number of layers of the network. When dealing with the vast majority of specific problems, the network structure consisting of three levels can basically deal with it. Therefore, when studying the purchase volume of cross-border e-commerce platforms in this paper, due to the small amount of data for each variable index, we choose to build a three-layer BP neural network model with only one hidden layer.
2. Enter the setting of the number of nodes in this layer. When getting a specific research question, the number of variables involved in the research object determines how many nodes to design in this layer. After analyzing the influencing factors of cross-border e-commerce platform procurement volume, this paper determines 13 influencing factors to model and analyze the cross-border e-commerce platform procurement volume, so this layer is designed as 13 nodes.
3. Output the setting of the number of nodes in this layer. For a practical research problem, when determining how many nodes to design in this layer, it should also be based on the actual situation. The problem studied in this paper is to predict the purchase volume of cross-border e-commerce platforms, so the number of nodes in this layer is set to 1.
4. Setting the number of hidden layer nodes. When constructing the network structure, the number of hidden layer nodes plays a very critical role. However, up to now, the research has found that there is still no ideal solution to the problem of how to select an appropriate number of hidden nodes for the hidden layer method, so this paper uses the empirical formula summarized by the researchers to determine the number of hidden layer nodes *m*.
5. Select the transfer function used in the network model. When constructing the network model in this paper, the Sigmoid function, which is widely used in the nonlinear transfer function, is selected because it has the characteristics that the linear function does not have, and its smoothness and differentiability make it occupy a special position in the transfer function. And it also has strong fault tolerance performance. The output layer directly selects a pure linear function.
6. Determine the method for training the network model and the parameters involved in the network model. In this paper, when using the training data to train the network model, a stochastic gradient descent (sgd) strategy, which is different from the standard BP algorithm, is selected. This algorithm randomly selects data with the same sample size for training, and then only uses this part of the sample data to calculate the gradient. In addition, set the number of epochs of model training (epochs) to 1000 and finally determine the learning rate of 0.09 and the momentum coefficient of 0.85 through multiple test adjustments.

**Figure 5:**
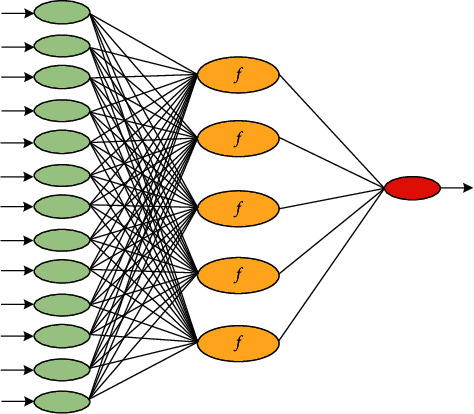
The network structure.

Based on the results of the above-determined parameter values, the BP neural network model is finally constructed, and the network structure is shown in Figure 5.

The study found that the input variables of the BP neural network in the previous month have a very close relationship with the input variables in the next month. The calculation results show the following rules: using the relevant factors of consumption behavior in the previous month as the input and the sales volume in the next month as the output, the model prediction values obtained from the training set and the test set have a high degree of fit, and the prediction is more accurate. Therefore, the data of November and December of each product are removed, and the output result of each data is moved up by one. That is, the input data of the previous month are used to match the output results of the next month. This also solves the problem that the unknown month cannot be obtained by the input variable. Due to insufficient inventory, some items have preorders in certain months. During data processing, the sales and preorders are aggregated, and the sum is calculated as the output result of this month.

**Figure 6:**
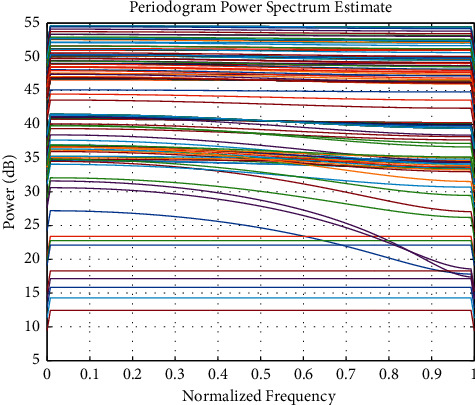
Normalized frequency.

This article uses the data from January 2017 to April 2019 as the training set and the data from May to August 2019 as the test set. After model calculation, the output result in September is the sales volume in October 2019, that is, the quantity that needs to be purchased in September. Since the purchase is made once a month, the goods purchased in September will arrive in October at the latest, which can also effectively reduce the problem of inventory backlog. The categories of predicted commodities are selected as food, cosmetics, and clothing. The normalized frequency is plotted in Figure 6.

## 4. Empirical Analysis

This section will perform data preprocessing on the raw data exported from the company database. Data preprocessing is the process of preprocessing dirty data such as outliers and missing values in the data set before building model features. The quality of data preprocessing directly affects the fit and robustness of the model. There are still many unreasonable parts in the current data set that need to be preprocessed before constructing features, including the processing of missing values and the smoothing of sales.

**Figure 7:**
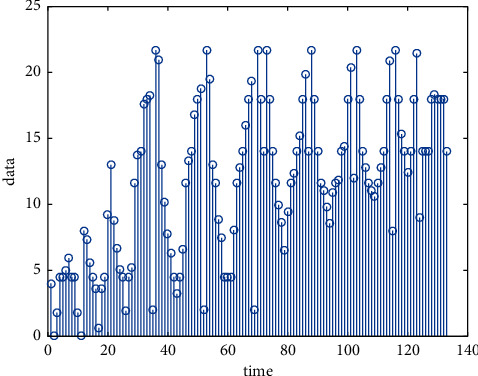
Data.

In the feature engineering of data mining, the processing methods of missing values mainly include discarding method and filling method. For features with more missing values, the discarding method is generally adopted directly, otherwise it may cause more noise, which will affect the model parameter offset; for the features with fewer missing values, the filling method is generally adopted. Generally, there are three main ways to fill missing values: one is to directly regard the missing value as an attribute feature; the other is to use a representative such as 0 or −1 that does not conflict with other attribute values; and the third is to use the mean, median, etc. to fill in; use the data above and below the missing value. The data are plotted in Figure 7.

According to the analysis of commodity information, in the current data set, there are 85.73% missing values in the seasonal attribute of commodity information, and this feature does not have much significance for the overall sales of commodities, so this feature is discarded. At the same time, the fifth-level, sixth-level, and seventh-level categories in the category features of commodity information have no value at all, and the method of discarding such features is directly used for processing, while the third-level category and the fourth-level category have relatively There are few missing values, indicating that the category division of some products has not been divided into four-level categories, and only the first few categories can be used to represent, so you can fill in these two features and other attribute values that do not conflict with −1 to handle missing values. And in the user performance data of the product and the date in the sales data of the product, there are some missing values. By comparing the upper and lower data of the missing value in the user performance data of the product, it is found that in the data with missing date, the date of the previous item, and the date of the next item are consecutive, indicating that these null data are unreasonable data generated by the program and should delete these data directly. In the commodity sales data, the dates in the data are continuous, and the missing date data can be directly filled with the date of the previous data.

Kalman smoother calculates the current optimal amount based on the current sales volume and the previous sales volume and “error” to smooth the time series sales, eliminate the interference of “noise,” and is conducive to the learning of the model. This article uses the KalmanFilter smooth function included in Python's pykalman library to smooth the sales data, but because the sales data ares both a feature and a predicted value, this article adds a feature column to the smoothed value to avoid affecting the authenticity of the predicted value.

Data normalization is to map feature variables between 0 and 1 according to a certain scaling ratio to avoid problems caused by different dimensions. According to the user behavior characteristic variables for the weekly statistics of products, the number of sku clicks is already two orders of magnitude difference between adding to the shopping cart and adding to the collection, and the value range of the discount is between 0 and 1. If the data are not normalized, normalization processing, the difference between different dimensions, will cause the model to be more affected by the features with larger values, so it is necessary to normalize the feature variables, and process the dimensional feature variables into dimensionless feature variables.

Use the Sklearn.preprocessing. MinMaxScaler function used to normalize 6 variables such as the number of clicks, the number of add-ons, the number of favorites, the average sales volume of the brand, the average sales volume of the category, and the historical sales volume of sku and save the maximum value of each feature value and minimum value. When the model is deployed online, the same measures are also taken for the corresponding feature variables in the new data.

**Figure 8:**
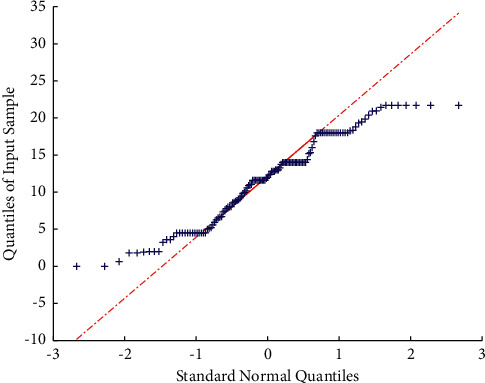
The results.

Using the data from 2017 to 2019 as the modeling basis, predict the subsequent data in 2020. According to the above learning steps, after thousands of times of learning, the squared error (input is the normalized sample) can be achieved. Judging from the learning speed, its speed can be compared with the commonly used BP learning algorithm. In order to verify whether the evolutionary strategy will fall into the local extreme point, 10 groups of weights were randomly selected for learning. For these 10 groups of weights, the evolutionary strategy can be successfully learned without falling into the local extreme point. Compared with the BP learning algorithm, which is easy to fall into local extreme points, it has great advantages. After learning, the network approximates all the sample points well, that is, the neural network has well realized the function mapping relationship between sales volume, price, and income. The neural network has good associative ability, so it should have good prediction ability for future sales. In order to verify this, the learned neural network is used to predict the purchase volume of cross-border e-commerce platforms after 2020. The results are shown in Figure 8. Curve in the figure is the actual purchase volume of the cross-border e-commerce platform, and the curve is the output of the neural network. It can be seen from the figure that the neural network can well approximate the training sample points (2019) and has strong predictive power (post-2020 data). It can be seen from the figure that the average relative error of the neural network is only 1.03%, indicating that it is feasible to build a sales forecast model with a neural network, and the forecast accuracy is quite high.

Through the above comparative analysis results, it is found that the prediction error of BP neural network is small, and the prediction accuracy is relatively high. Therefore, with the help of this model, based on the data of each variable index from 2017 to 2019, the purchase volume of cross-border e-commerce platforms after 2020 is predicted and estimated. Input the standardized data of each variable index in 2018 into the already constructed cross-border e-commerce platform purchase volume prediction model (i.e, BP neural network prediction model) and finally calculate that the cross-border e-commerce platform purchase volume in the next year is approximately 1,069,940. The relative error between the sales volume predicted by the model and the actual sales volume is 1.03%. Since the prediction results of the article using the BP neural network model are based on the assumption that the country has not issued major policies for the e-commerce market and the e-commerce companies have not changed significantly, the predicted results have certain limitations, but the model the forecast results can be referenced by the power supplier company.

**Figure 9:**
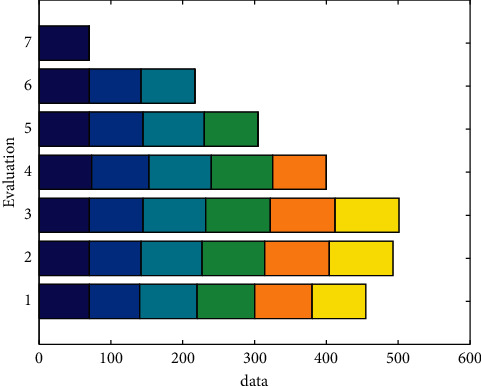
Evaluation.

Based on various modeling analysis results, it can be found that the BP neural network prediction model has the advantages of high prediction accuracy and relatively accurate prediction results. Therefore, compared with the linear regression forecasting model, the BP neural network model has a broad prospect in the field of sales forecasting with many influencing factors. This model can provide an effective auxiliary means for e-commerce companies to forecast and estimate sales. E-commerce companies can use the data analysis method of BP neural network to forecast and estimate future sales, and then companies can make corresponding effective strategies with reference to the forecast results. The evaluation is shown in Figure 9.

## 5. Conclusion

This paper predicts the purchase volume of goods on an e-commerce platform. In the research, the time series characteristics of commodity sales are analyzed, and combined with commodity consumption behavior, the prediction application of BP-based neural network in cross-border e-commerce platform is proposed. Through analysis, irrelevant factors were removed, and corresponding data processing was done. Afterwards, the analysis and comparison of predictions are made with various models, and it is concluded that this method can better solve the problem of predicting the purchase volume of the platform. After verification, the average error percentage of this method is 5.9%, and the method can be extended to the practical application of enterprises.

## Data Availability

The data set used in this study can be accessed upon request.
